# Selective laser melting of high-performance pure tungsten: parameter design, densification behavior and mechanical properties

**DOI:** 10.1080/14686996.2018.1455154

**Published:** 2018-04-18

**Authors:** Chaolin Tan, Kesong Zhou, Wenyou Ma, Bonnie Attard, Panpan Zhang, Tongchun Kuang

**Affiliations:** a School of Materials Science and Engineering, South China University of Technology, Guangzhou, China; b National Engineering Laboratory for Modern Materials Surface Engineering Technology, Guangdong Institute of New Materials, Guangzhou, China; c School of Metallurgy & Materials, University of Birmingham, Birmingham, UK

**Keywords:** Additive manufacturing, selective laser melting, tungsten, refractory metal, parameter design, densification, linear energy, laser parameter, molten pool, property, 10 Engineering and Structural materials, 303 Mechanical / Physical processing, 210 Thermoelectronics / Thermal transport / insulators, 106 Metallic materials, 305 Plasma / Laser processing

## Abstract

Selective laser melting (SLM) additive manufacturing of pure tungsten encounters nearly all intractable difficulties of SLM metals fields due to its intrinsic properties. The key factors, including powder characteristics, layer thickness, and laser parameters of SLM high density tungsten are elucidated and discussed in detail. The main parameters were designed from theoretical calculations prior to the SLM process and experimentally optimized. Pure tungsten products with a density of 19.01 g/cm^3^ (98.50% theoretical density) were produced using SLM with the optimized processing parameters. A high density microstructure is formed without significant balling or macrocracks. The formation mechanisms for pores and the densification behaviors are systematically elucidated. Electron backscattered diffraction analysis confirms that the columnar grains stretch across several layers and parallel to the maximum temperature gradient, which can ensure good bonding between the layers. The mechanical properties of the SLM-produced tungsten are comparable to that produced by the conventional fabrication methods, with hardness values exceeding 460 HV_0.05_ and an ultimate compressive strength of about 1 GPa. This finding offers new potential applications of refractory metals in additive manufacturing.

## Graphical abstract

**Figure:**
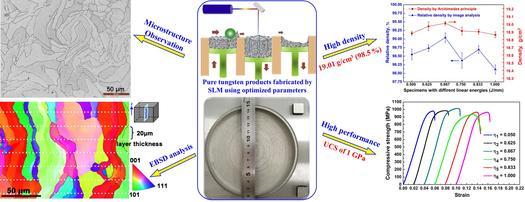

## 1. Introduction

Tungsten (W), as the highest melting point refractory metal, has many unique physical and chemical properties, including high density, high thermal conductivity, high recrystallization temperature, low thermal expansion, and high strength and hardness at room and elevated temperatures. Tungsten and its alloys have been applied in numerous fields, including as lighting engineering, electronics, manufacturing, aerospace, military, medical field, and especially nuclear field [1–4]. For example, tungsten is of interest to the nuclear industry as a promising candidate for plasma-facing materials (PFM) in future nuclear fusion devices such as the International Thermonuclear Experimental Reactor (ITER) and high-performance rocket nozzles, as intensive transient heat loads must be withstood alongside the requirements of limited tritium retention, and handling enormous particle ﬂux of hydrogen, helium and neutrons [1,5,6].

However, due to its relatively high ductile-brittle transition temperature, tungsten products are conventionally manufactured through powder metallurgy (PM), spark plasma sintering (SPS), chemical vapor deposition (CVD), hot isostatic pressing (HIP), and thermoplastic routes [7,8]. Nevertheless, these methods have limitations in producing parts with complex internal and external structures. Therefore, a new approach of producing tungsten components is necessary.

Additive manufacturing using Selective laser melting (SLM) is capable of producing 3D parts in an incremental layer-by-layer manner using the laser to melt, sinter, and bond powder particles together in a thin layer on a powder bed [9,10]. Due to many irreplaceable superiorities such as high resolution and dimensional tolerance, high material and resource efficiency, good part design and production flexibility, desirable mechanical performance of SLM technology [11–13], it has been gradually applied in customized medical and dental application fields, tooling inserts with conformal cooling channels and functional components with high geometrical complexity such as porous and lattice constructs [14–16].

Materials well suited to SLM have good laser absorption and balanced properties of melting point, thermal conductivity, surface tension and viscosity [5]. SLM of pure tungsten encounters nearly all intractable difficulties of metal SLM manufacture. The high melting point (3695 K) of the element results in high cohesive energy, while its high surface tension (2.361 N·m^−1^) promotes the formation of balling phenomenon and its high thermal conductivity (173 W·m^−1^ k^−1^) leads to rapid solidification and cooling. The high viscosity (about 8 × 10^−3^ Pa·s), which derives from the high intrinsic cohesive energy, significantly reduces the flowability of the molten pool [5]. The oxidation sensitivity may lower wettability and lead to the formation of cracks when even the small amounts of oxygen are absorbed by the molten pools.

Due to the aforementioned difficulties, quite a few works concerning additive manufacturing tungsten were reported, and the high-density components have still not yet been obtained [5,17]. Deprez et al. [17] produced a complex collimator from pure tungsten powder using SLM, achieving a density of 17.31 g/cm^3^ (89.92 of theoretical density). Zhou et al. [5] investigated the balling phenomena in selective laser melted tungsten, obtaining a specimen density of 16 g/cm^3^ (82.9% of theoretical density). Both of them were unable to produce high density tungsten parts and characterize the mechanical performance. In our work, high density pure tungsten parts were produced by SLM through optimization of the processing parameters. The densification, microstructure, and mechanical performances were characterized in detail. The results demonstrate that even the highest melting point metals can be produced via additive manufacturing, expanding the potential applications of this technique.

## 2. Experimental details

### 2.1. Materials and SLM process

The raw material used in this study was high purity, plasma spheroidized tungsten powder (purity 99.9%) supplied by Tekna Advanced Materials Inc. (Quebec, Canada). The size distribution of the powder was measured by a HORIBA Partica LA960 laser scattering particle size analyzer (Tokyo, Japan), and the trace elements (carbon, oxygen, and nitrogen) were estimated by an Elementar Vario EL CUBE elemental analyzer (Langenselbold, Germany).

The experiments were carried out in an EOS M290 SLM system (EOS GmbH, Krailling, Germany). The laser parameters were orthogonally designed in which the laser power (*P*) and scanning speed (*v*) were set in the range of 200–370 W and 100–400 mm/s, respectively. The linear energy was defined as *η* = *P*/*v* (J/mm). The laser scanned in a zigzag pattern with 67° rotation between adjacent layers to minimize the residual stress. When selecting the required layer thickness, a balance must be reached between achieving a fine resolution and allowing for good powder flowability. This usually occurs in the range of 20–100 μm [18]. A layer thickness of 20 μm was selected as it allows for the complete melting of the powder and enables good bonding between the layers, since an increase in thickness would cut down the amount of energy reaching the underlying layer, and reduce the thermal penetration depth for the re-melting of the underlying layers. Incomplete melting or poor bonding could result in regions of greater inhomogeneity with a higher prevalence of cracks and incomplete fusion in the material [19]. A larger layer thickness could also lead to insufficient volumetric laser energy density, which promotes balling due to a lack of wetting of the molten pool with the previously deposited layer [20]. Tungsten powder has an extremely high melting point and is prone to balling during SLM, thus selecting the correct layer thickness is crucial. Prior to SLM processing, the building platform was pre-heated to 50 °C (323 K). The oxygen content in the process chamber was kept below 0.1 vol. % through a continuous flow of argon, as balling can be significantly reduced by improved oxygen control in the process chamber [18,20].

### 2.2. Characterization

The roughness, *S*_*a*_, of the fresh surfaces for the as-fabricated specimens was evaluated by a BMT SMS Expert 3D model optical profilometer (Breitmeier Messtechnik GmbH, Ettlingen, Germany) with a measuring area of 2 mm × 2 mm, the average values and standard deviations were estimated from 5 measurements. The density of the as-fabricated tungsten specimens was determined by both image analysis method using a Leica DMI5000M (Wetzlar, Germany) optical microscopy (OM) and the Archimedes method. The image analysis method evaluates the porosity within a specimen by calculating the percentage area of porosity on the polished surfaces, while the Archimedes method calculates the specimen’s density according to ASTM B962-08. The microstructural and surface morphology observations were performed using the OM and a Zeiss Merlin (Jena, Germany) ﬁeld emission scanning electron microscope (FE-SEM). Prior to microstructural observations specimens were etched in standard Murakami’s solution (1:1:10 for KOH: K_3_[Fe(CN)_6_]: H_2_O). X-ray diffraction (XRD) was conducted using a Bruker D8 Advance Diffractometer (Karlsruhe, Germany) with a Cu Kα radiation (wavelength, *λ* = 0.15418 nm), at 40 kV and 40 mA in a 2*θ* range of 30–90° using a step size of 0.02°. Electron backscattered diffraction (EBSD) was carried out on a Hitachi S-3400N SEM system (Tokyo, Japan) at 20 kV through an integrated Oxford/HKL EBSD detector, using a step size of 300 nm. Specimens for EBSD test were mirror polished and then vibratory polished for 5 h in order to remove any scratches and plastic deformation from the surface layer. The EBSD data was analyzed using Channel 5 software (HKL Technology, Inc., Connecticut, CA, USA). Micro-hardness of the specimens was measured by a Shimadzu HMV-2T micro Vickers tester (Tokyo, Japan) with a load of 50 gf for 10 s. All hardness indentations were applied to the polished horizontal (parallel to the build plate) surfaces, and an average value and corresponding standard deviation were estimated from 10 measurements. The compressive tests for the as-fabricated tungsten specimens were carried out on a SANS CMT 5105 universal material testing machine (MTS Systems China Co. Ltd., Shenzhen, China) with a strain rate range of 3.5 × 10^−3^/s–4.5 × 10^−3^/s. The length to diameter (*L/D*) ratio of the specimens was 3 (as per ASTM E9-09), and all the specimens were simultaneously polished by a grinding machine to ensure identical surface quality.

## 3. Results and discussion

### 3.1. Parameters design and analysis

#### 3.1.1. Powders

**Figure 1.**
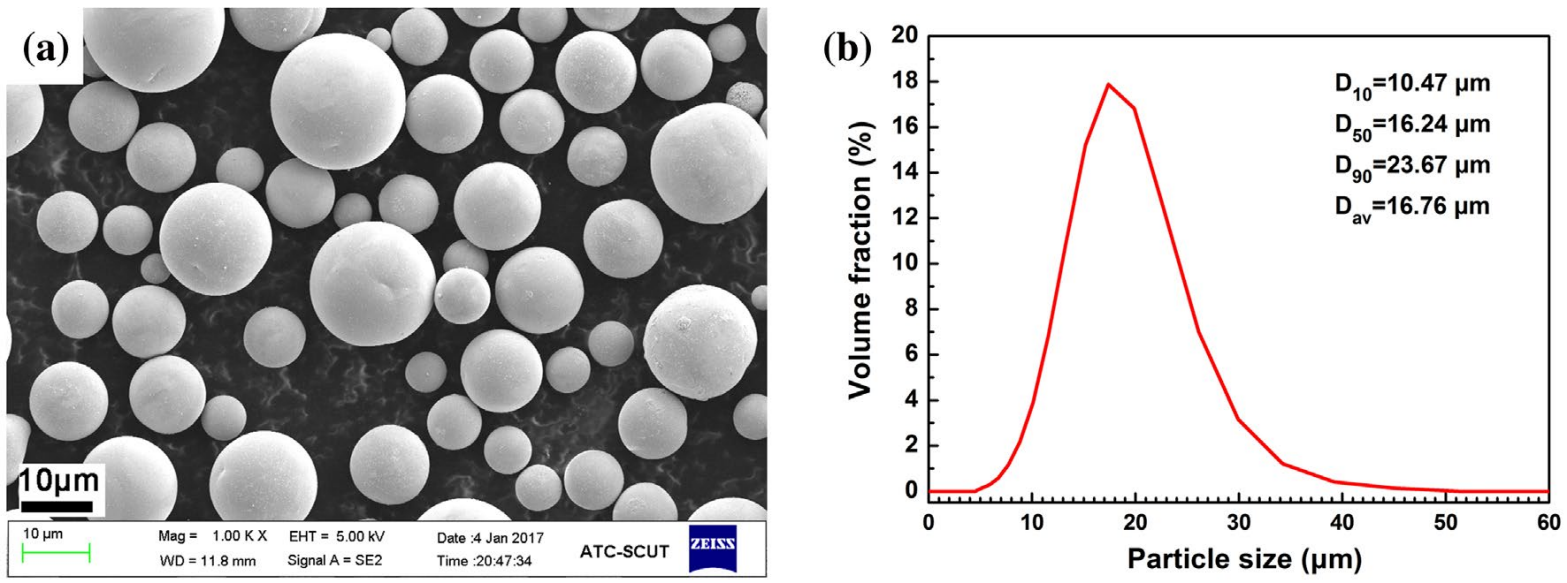
SEM morphology (a) and size distribution (b) of the pure tungsten powder.

In addition to laser parameters, powder morphology and size distribution also have important effects on the SLM process. The morphology of the tungsten is of fundamental importance. As such a highly spherical powder was chosen, to improve the flowability, and promote the wetting between the melt pool and powder particles. Zhou et al. [5] produced pure tungsten by SLM using powder with an irregular morphology, which may have been one of the factors resulting in lower part density (16 g/cm^3^) obtained in their study. Wang et al. [21] demonstrated that spherical powder could increase laser absorptivity and packing density in comparison to polyhedral powders. In addition to this, the size distribution of the powder also plays an important role during the SLM process. As depicted in Figure 1, the tungsten powder was carefully selected with a relatively small mean particle size of 17 ± 5 μm, and a narrow powder size distribution, with D_50_ and D_90_ values of 16.24 and 23.67 μm, respectively. The levels of the main impurity elements O, C, N in the powders are measured to be 220, 580, and 470 ppm, respectively. The comparatively low oxygen content in the tungsten powder may also suppress the balling tendency during SLM.

The temperature of the powder bed is increased as it absorbs the laser energy $E_{\text{laser}}$, as expressed in the following heat balance equation [22]:

$$E_{\text{laser}}=\frac{4}{3}{\pi r}_{p}^{3}\rho C_{p}\Delta T$$

where *r*_*p*_, ρ, $C_{p}$ and Δ*T* are the particle radius, density, specific heat and temperature increase in the powder particles, respectively. Obviously, the smaller particles are more easily heated due to the lower thermal capacitance. Nevertheless, significantly undersized particles agglomerate more easily due to Van der Waals forces, reducing powder flowability and causing poor powder deposition [18].

#### 3.1.2. Laser parameters

Theoretically, an estimate of the energy required to melt a volume of material *Q*_*p*_ (J/mm^3^), can be determined from the expression [23]:

$$Q_{p}=\rho C_{p}\left(T_{m}-T_{o}\right)+\rho L_{f}$$

where *ρ* is the density (kg m^−3^) of bulk material, *C*_*p*_ is the specific heat (J·kg^−1^ K^−1^), *T*_*m*_ is the melting point (K), *T*_0_ is the initial temperature (K), and *L*_*f*_ is the latent heat of fusion (KJ·kg^−1^). The detailed values of these physical parameters are listed in Table 1, the energy required to melt of pure tungsten powder preheated to 323 K is calculated to be about 8.595 J/mm^3^.

**Table 1.** Physical parameters used for theoretical calculation in laser parameters design.

Physical parameter	Unit	Value
Density/*ρ*	kg·m^−3^	19.30 × 10^3^
Speciﬁc heat/*C*_*p*_	J (kg·K)^-1^	132
Melting point/*T*_*m*_	K	3695
Initial temperature/*T*_*o*_	K	323
Latent heat of fusion/*L*_*f*_	J·kg^−1^	2.20 × 10^5^
Laser absorptivity/*α*	–	0.41
Laser beam radius/*r*_*b*_	mm	50 × 10^−3^
Laser melting radius/*r*	mm	100 × 10^−3^
Melting zone depth/H	mm	30 × 10^−3^

**Figure 2.**
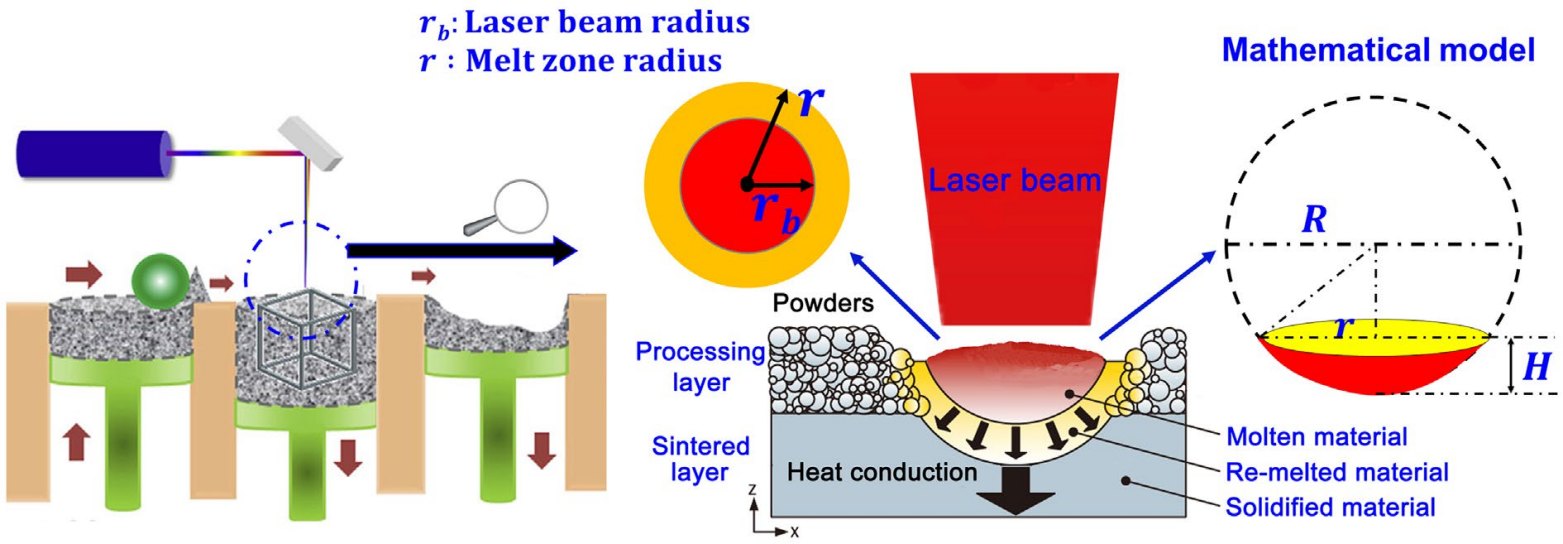
Schematic diagram of the SLM process and the heat transfer in molten pool.

Figure 2 illustrates the SLM process and the heat transfer in the molten pool. The intensity profile of the laser beam is assumed to have a Gaussian distribution. The laser energy flux can therefore be expressed as [24]:

$$E\;\left(r\right)=\frac{2\alpha P}{\pi r_{b}^{2}}\text{exp}\left(-\frac{2r^{2}}{r_{b}^{2}}\right)$$

where *P* is the laser power, *r*_*b*_ is the laser beam spot radius, *r* is the distance from the beam center, and *α* is absorptivity coefficient. The average laser energy flux $E_{s}\left(\text{W}/{\text{mm}}^{2}\right)$ can be expressed as:

$$E_{s}=\frac{1}{\pi r_{b}^{2}}\int_{0}^{r_{b}}2\pi rE\left(r\right)\text{d}r=\frac{2\alpha P}{\pi r_{b}^{2}}\left(1-e^{-2}\right)$$

The energy loss in the molten pool can be divided into three parts, including convective heat loss, radiation heat loss, and heat loss through evaporation [25]. Usually, the heat loss due to convection and radiation is generally assumed to be about 10% [24,26]. Previous calculations revealed that the latent heat of evaporation is relatively large, causing prominent energy loss, especially at high temperatures. The heat loss through evaporation was therefore estimated to be 10% [25]. Therefore, the total heat loss during the SLM process was assumed to be 20%. This estimation of heat loss is of a similar level with previous work of direct laser deposition H13 tool steel, in which about 75–85% of original power reached the molten pool surface [27]. Hence, the effective laser energy flux $E_{\text{in}}\left(\text{W}/{\text{mm}}^{2}\right)$ used for melting the powders is:

$$E_{\text{in}}=E_{s}-E_{\text{loss}}=E_{s}\;\left(1-0.2\right)$$

Therefore, the volumetric laser energy per unit volume $Q_{v}\;\left(\text{J}/{\text{mm}}^{3}\right)$ of tungsten powders can be expressed as

$$Q_{v}=\frac{\pi r_{d}^{2}E_{\text{in}}\Delta t}{V_{m}}\;$$

$$\Delta t=\frac{2r_{b}}{v}$$

where Δ*t* is the laser exposure time, *v* is the laser scan speed, *V*_*m*_ is the effective volume of molten pool. As shown in Figure 2, we simplified the profile of the molten pool as a part of sphere, and the *V*_*m*_ can be estimated as:

$$V_{m}≅\frac{1}{2}\times \frac{4}{3}\pi R^{3}-\frac{1}{3}\pi \left(\text{R}-\text{H}\right)\left(R^{2}+r^{2}+\text{Rr}\right)$$

where *r* is the effective radius of laser beam, H is the effective laser melting depth and *R* is the radius of the sphere. Normally, the width of the laser melting track on the powder is about twice as the radius of laser beam *r*_*b*_, so we suppose *r* = 2*r*_*b*_. Besides, for the purpose of introducing laser re-melting of the under layer, we designed the effective laser melting depth to be as thick as two layer thickness (i.e. H = 40 μm). Since, it enables a reliable connection between the layers and suppresses the balling phenomenon as balling can occur when tungsten molten pools solidify too quickly to spread out completely. Furthermore, choosing a suitable laser re-melting depth would also reduce the tendency of cracking, since re-melting can break up the oxide films and reduce the thermal stress [5,18,20,28]. Consequently, *V*_*m*_ and *Q*_*v*_ can be rationally calculated. When $Q_{v}\geq Q_{p}\left(Q_{p}=8.595\;\text{J}/{\text{mm}}^{3}\right)$, the laser should theoretically completely melt the powders. To meet the requirement of $Q_{v}\geq Q_{p}$, the condition for linear energy η≥0.42J/mm needs to be satisfied, therefore, we designed the minimal *η* to be of 0.5 J/mm, in which the *P*, *v* and *Q*_*v*_ are 200 W, 400 mm/s and 10.287 J/mm^3^, respectively. A plenty of laser parameters were orthogonally designed with *P*, *v* and *η* setting in the range of 200–370 W, 100–400 mm/s, and 0.5–3.7, respectively.

### 3.2. Parameters optimization

**Figure 3.**
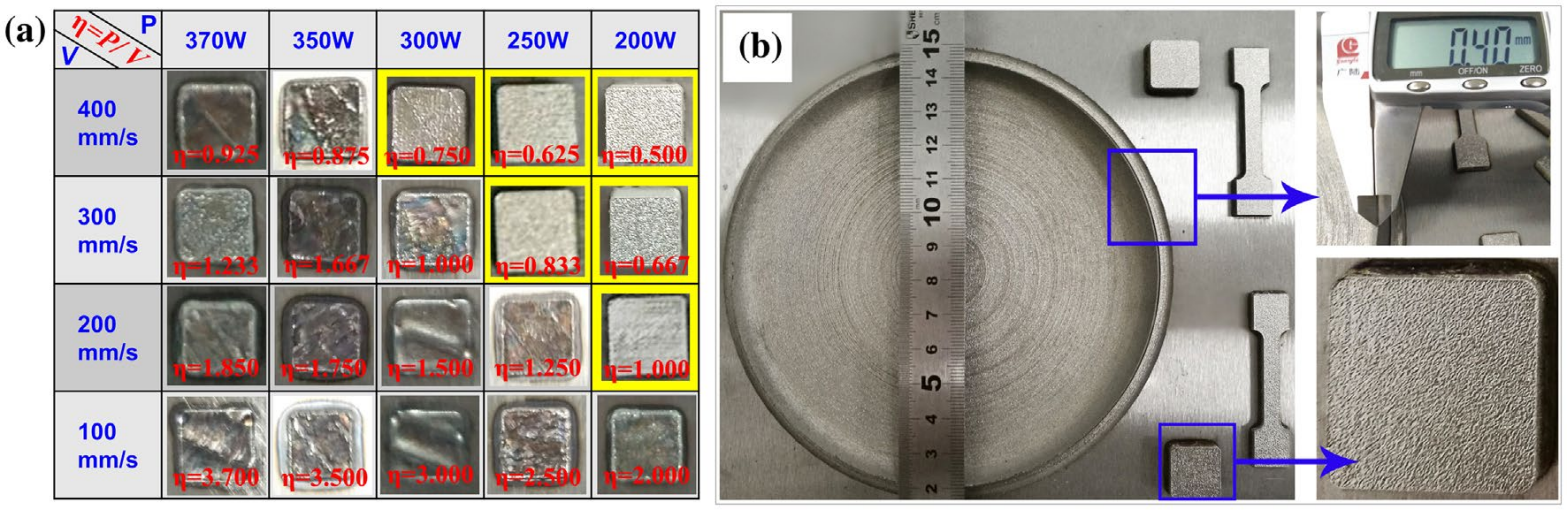
Optical images of SLM fabricated pure tungsten parts: (a) blocks fabricated with different linear energies; (b) 0.40 mm thin-wall tungsten part and specimens produced with *η*_3_ = 0.667 J/mm.

Figure 3 shows the optical images of SLM-produced pure tungsten parts. Figure 3(a) reveals the relationships between the sintering formability and the laser parameters, where the specimens produced with *η*_1_ = 0.500, *η*_2_ = 0.625, *η*_3_ = 0.667, *η*_4_ = 0.750, *η*_5_ = 0.833 and *η*_6_ = 1.000 J/mm (marked in yellow background color) present better sintering formability. Thus, the parameter window to obtain a good, structurally sound component ranges between *η* values of 0.5–1.0 J/mm, with the corresponding *P* and *v* covering the range of 200–300 W and 200–400 mm/s, respectively. Further increasing the linear energy leads to burning loss and surface corrugation, which destroyed the forming quality and hindered the powder recoating. Besides, even the same linear energy, the different combination between *P* and *v* presented distinct forming qualities, for example, when the linear energy was in the same value of *η* = 1.0 J/mm, the specimens prepared with *p* = 200 W and *v* = 200 mm/s show better sintering formability than these with *p* = 300 W and *v* = 300 mm/s. This is because the lower speed allowed more time for pools wetting and spreading, which compensated the intrinsic low flowability of tungsten. Figure 3(b) shows the optical images of an SLM-produced thin-wall (0.4 mm in thickness) tungsten part and some specimens with the *η*_3_ linear energy. The shining and smooth surface without any macrocracks and balling certifies reasonable laser parameters and high sintering quality.

### 3.3. Surface morphology characterization

**Figure 4.**
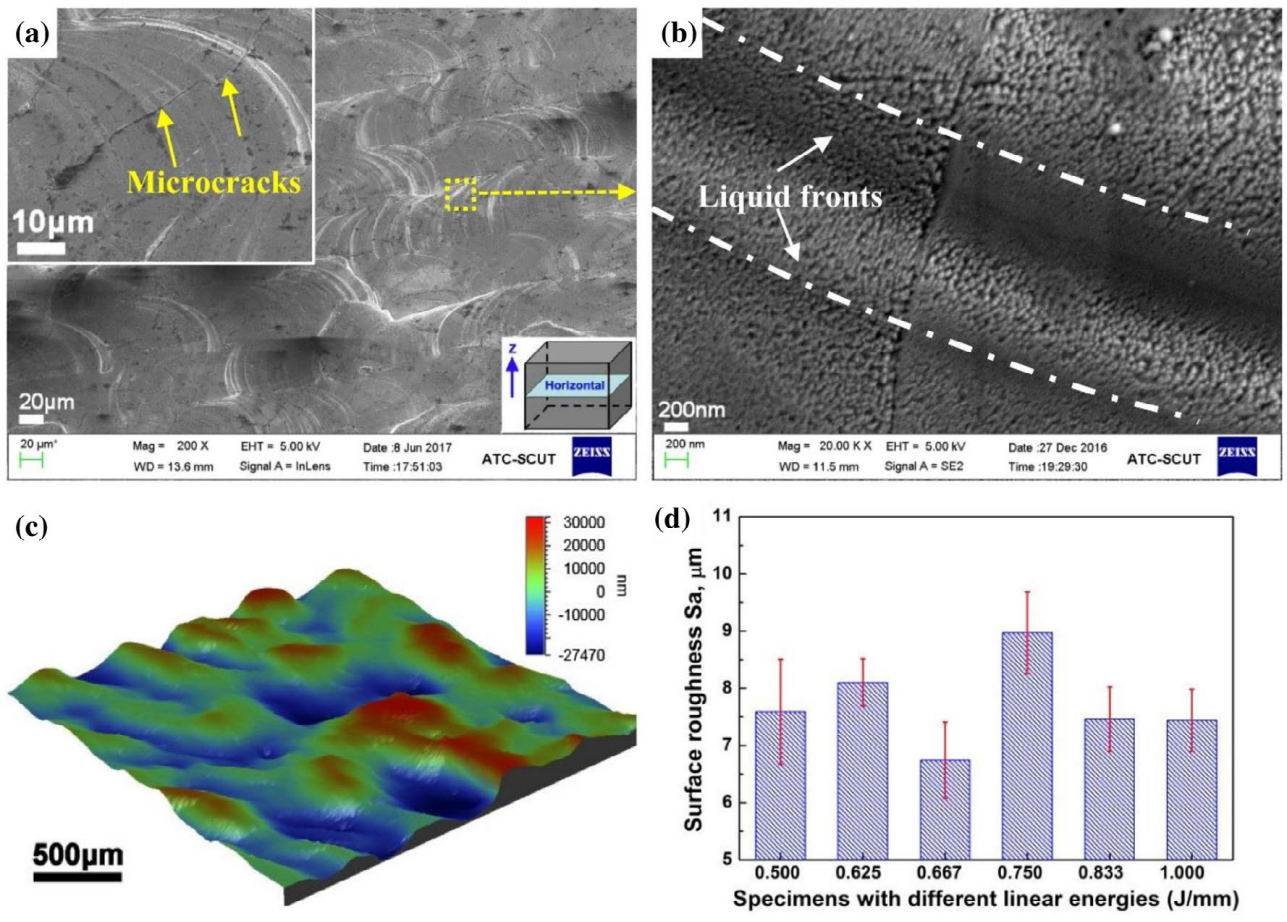
Surface morphology and roughness taken from horizontal surfaces of SLM fabricated pure tungsten: (a) SEM image showing regular laser tracks (*η*_3_ = 0.667 J/mm) and microcracks (inset image); (b) corresponding high-magnification SEM image showing massive nanocrystals in the liquid fronts; (c) the corresponding 3D topography image of the untreated fresh surface and (d) relationships between surface roughness and linear energy summarized from the 3D topography images.

Figure 4 shows the untreated fresh surface morphology and roughness of SLM fabricated pure tungsten taken from horizontal cross-sections. A relatively smooth and dense surface with regular liquid fronts is obtained and exhibited in Figure 4(a). No balling or individual unmelted particles can be observed, showing that all the laser tracks have good metallurgical bonding with each other excepting some scattered black slags. Microcracks can also be observed through the inset high-magnification SEM image in Figure 4(a). Normally, cracks in SLM processed parts are caused by thermal residual stresses, which arise from two mechanisms, i.e. thermal gradient mechanism (TGM) and the cool-down phase of molten top layers [29]. Additionally, high brittleness and insufficient plasticity of pure tungsten also contribute to the formation of cracks. High-magnification SEM observation on the liquid fronts is provided in Figure 4(b), massive nanosized columnar crystals are perpendicular to the fronts moving directions, suggesting an extremely high cooling rate of molten pools. In fact, the ultra-high cooling rate of laser processing could reach the order of 10^10^ K/s [30], and the relative high thermal conductivity (173 W·m^−1^ K^−1^) also contributes to the high cooling rate and grain refinement.

Surface roughness is another important factor reflecting the sintering formability. The representative (*η*_3_ = 0.667 J/mm) 3D topography image of surface is provided in Figure 4(c), which also reveals that a relativly smooth surface is obtained. Relationships between the average surface roughness and the linear energy are depicted in Figure 4(d), which was summarized from a number of 3D topography analyses. The average S_a_ of the original surface of the specimens is in the range of about 7–9 μm, with the highest value of 8.97 μm in the *η*_4_ specimen and the lowest value of 6.74 μm in the *η*_3_ specimen. The relative smooth surface implies the high formation qualities.

### 3.4. Densiﬁcation behavior

**Figure 5.**
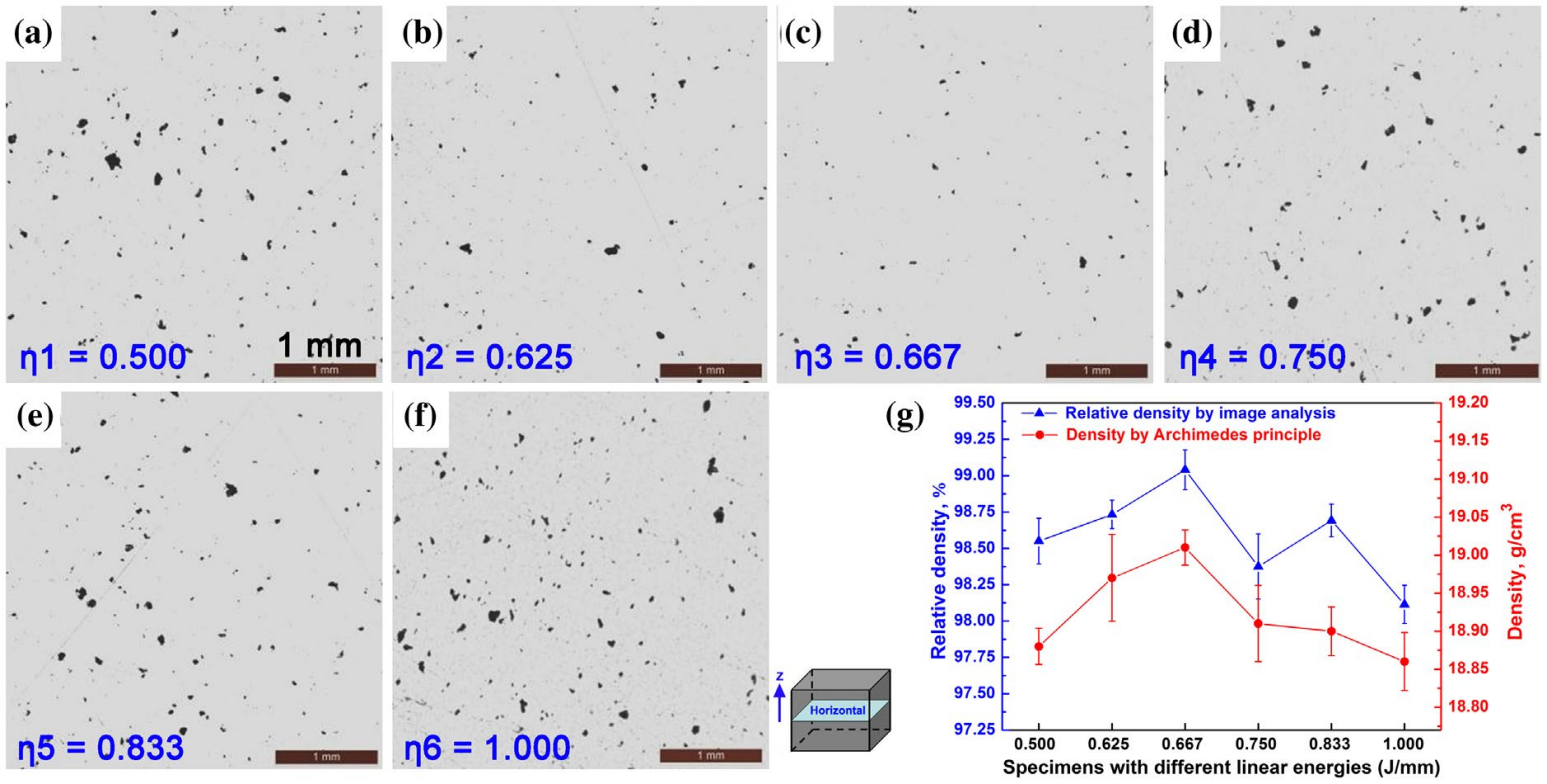
Optical micrographs showing the pores in the horizontal cross-sections of pure tungsten produced by SLM with different linear energies (J/mm): (a) *η*_1_ = 0.500, (b) *η*_2_ = 0.625, (c) *η*_3_ = 0.667, (d) *η*_4_ = 0.750, (e) *η*_5_ = 0.833, (f) *η*_6_ = 1.000; (g) density graphs obtained from Archimedes and image analysis methods.

Figure 5(a)–(f) show the representative polished OM images taken from the horizontal cross sections of specimens produced with *η*_1_–*η*_6_. A plenty of pores can be observed in *η*_1_ and *η*_6_ specimens, while relativly fewer micropores presented in *η*_3_ specimen. The relationship between *η* and density measured by both image analysis method and Archimedes method is shown in Figure 5(g). The relative densities measured by the image analysis method are about 98–99%, while the densities measured by Archimedes method are 18.86–19.01 g/cm^3^, which is 97.72–98.50% density of pure tungsten (19.30 g/cm^3^). The small but systematic difference of density estimated from images and Archimedes methods might originate from open pores. Despite this minor difference between these two methods, both results show the SLM-produced specimens achieved a high density of more than 97.5% for all specimens. Both curves show that an optimum density obtained at *η*_3_. Moreover, there is a decline tendency of density when the *η* value decreases from *η*_3_ to *η*_1_ or increases from *η*_3_ to *η*_6_, as also revealed in Figure 5(a)–(f).

There are two main reasons accounting for pores and densification behavior. First, turbulent flow of molten pools is incited by the high-energy density laser irradiation, and the pores are easily formed due to the protective gas rolled in the molten pools. On the one hand, this could be explained by the dynamic viscosity (*η*_*v*_) of the molten pool, which is defined as [31]:

$$\eta_{v}=\frac{16}{15}\sqrt{\frac{m}{\mathrm{kT}}}\;\sigma$$

where *m*, *k*, *T*, and σ are the atomic mass, Boltzmann constant, molten pool temperature and surface tension, respectively. The *σ* of tungsten is in negative linear relation with *T*, so increasing *T* can lower *σ* and lead to a decrease in *η*_*v*_ as a result. With the linear energy *η* increase from *η*_1_ to *η*_3_, the *T* of molten pools increases and the dynamic viscosity *η*_*v*_ decreases, which increases the flowability of molten pool and improves the density in return. On the other hand, the molten pool is significantly influenced by the dynamics of the Marangoni effects. Marangoni flow increases with the laser energy input, this increases the gas dragging probability toward the molten pool which in turn leads to pores formation in the solidified molten pool [32]. Therefore, increasing *η* from *η*_3_ to *η*_6_ caused the aggrandized porosity.

Second, the micropores may be ascribed to the balling phenomenon. During SLM process of pure tungsten, the droplet spread time (86.3 μs) is nearly twice as its solidification time (46 μs) [5]. The solidification front grows too fast, whereas the spreading time is limited. So the melt droplets could be arrested and solidify as globular islands, which severely hinders the uniform subsequent powder deposition. When the laser melts such an uneven powder layer, the movement of molten pool front undergoes a significant disturbance and even interruption. Consequently, it is difficult to completely fill the inter ball pores on the surface of the previous layer, leading to the interlayer pores and a limited densification [33]. Insufficient laser energy may cause the decrease in solidification time and enhance balling; moreover, the formation of incomplete melted powders in the molten pool could decreases the flowability and wettability, which can also increase the porosity. Therefore, the porosity increases with *η* decreasing from *η*_3_ to *η*_1_. Pores of SLM-produced pure tungsten are inevitable due to its intrinsic materials properties. It is worth mentioning that, the titanium and steel are easy to be produced by SLM, because the solidification time increases significantly with the increase of the melting temperature. The solidification time can exceed the spreading time at the temperature of around 2100 K for both Ti and Fe [5], which ensures the complete spreading of the melt droplets. So an almost fully dense part of Ti or Fe can be easily achieved, while pores of SLM-produced pure tungsten are inevitable in contrast. In this work, the careful analysis and control of the laser parameters allowed achieving densities of up to 19.01 ± 0.02 g/cm^3^ (98.50 ± 0.12% of theoretical density), which is much higher than the previous reported results of 16 g/cm^3^ (82.9%) [5], 17.31 g/cm^3^ (89.92%) [17], and 18.53 g/cm^3^ (96.01%) [21] for SLM-produced tungsten.

### 3.5. Microstructural observation and analysis

**Figure 6.**
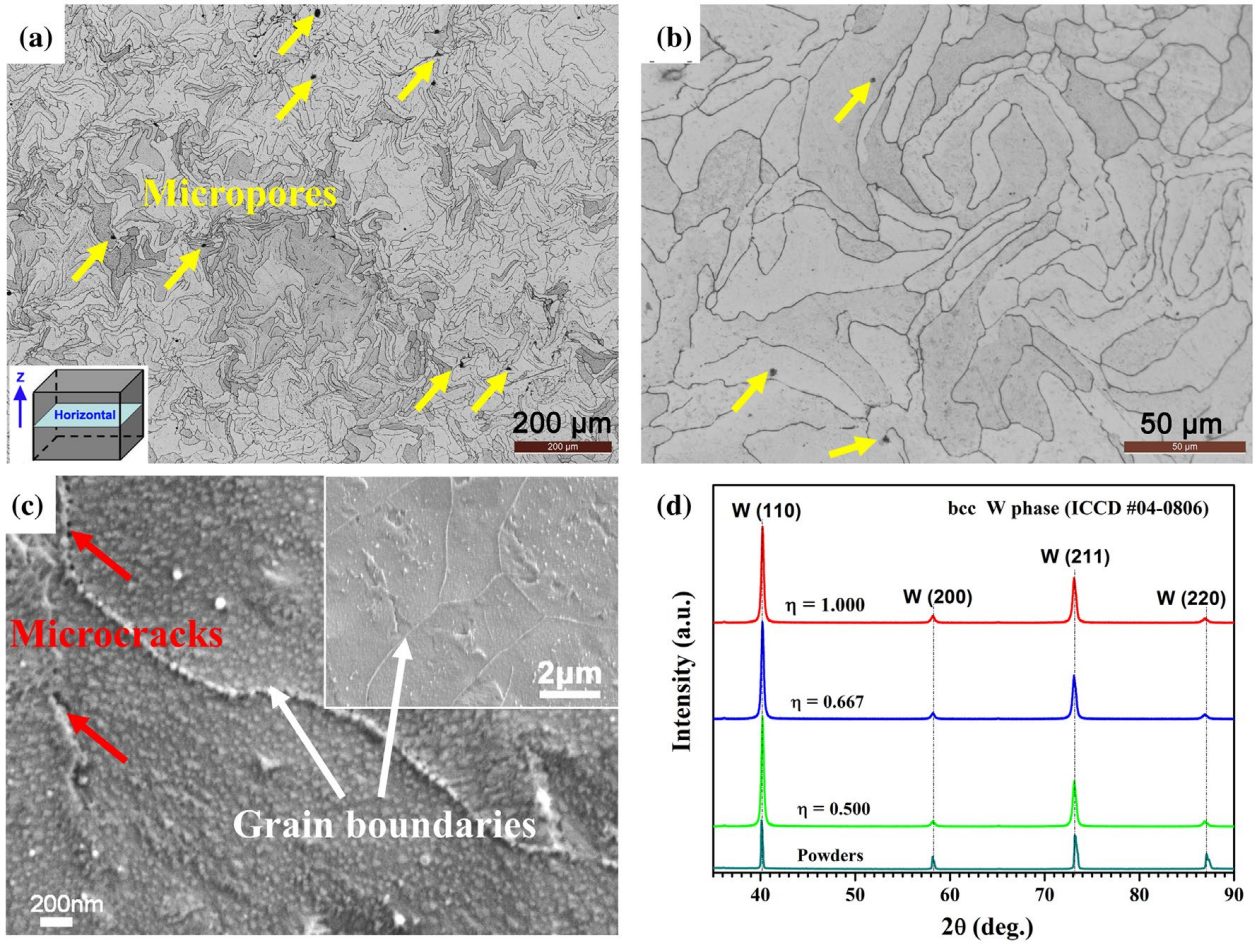
Microstructural observation and phase identification taken from horizontal cross-sections of SLM-produced pure tungsten: (a) low-magnification and (b) high-magnification OM macrographs; (c) low-magnification (inset) and high-magnification SEM morphologies, and (d) XRD patterns of powder and SLM-produced specimens.

Figure 6 shows the microstructural morphologies and XRD patterns taken from horizontal cross-sections of SLM-produced pure tungsten. The dense microstructure along with a few micropores was observed by OM and provided in Figure 6(a) and (b). The spherical pores are normally caused by gas entrapment in the molten pool, as discussed in Section 3.4. Grain boundaries could be clearly observed from the inset SEM morphology in Figure 6(c), and massive nanosized spot-like subgrains present inside the grains in high-magnification SEM image. The nanosized subgrains would form in response to rapid solidification with extremely high cooling rates present in the laser process [34]. Besides, as labeled in Figure 6(c), a few thermal cracks near or in the grain boundary may be caused by the thermal residual stress. Their formation was ascribed to the aforementioned tensile stress. In addition, the intrinsic properties of tungsten such as high brittleness, high oxidation sensitivity and low wettability also promote the formation of such cracks.

Figure 6(d) shows XRD patterns of raw tungsten powder and SLM-produced tungsten parts. All the diffraction peaks characterize the standard body-centered-cubic (bcc) tungsten phase (ICDD #04-0806) in (1 1 0), (2 0 0), (2 1 1), and (2 2 0) planes. Besides, the diffraction patterns of the SLM-produced specimens show wider peaks in comparison to those of the precursor powder. According to the Debye-Scherrer formula, this peak broadening is related to the small size of crystalline domains formed during the rapid solidification after laser processing; it may also indicate residual stresses within the SLM-produced specimens caused by lattice distortions [35,36].

**Figure 7.**
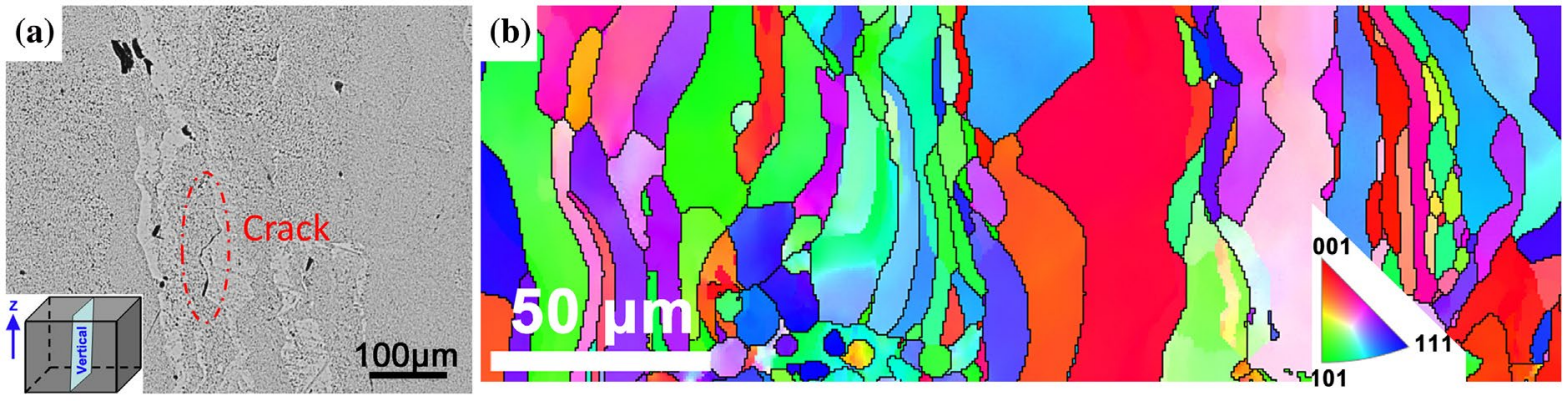
SEM and EBSD analysis carried along the building direction (Z direction): (a) SEM image showing a crack along building direction and (b) Inverse pole figure showing the microstructure and grain orientation map of SLM-produced tungsten.

As shown in Figure 7, SEM morphology and EBSD maps recorded in the middle region along the building direction (*Z* direction) were further performed to study the microstructure and texture development of as-fabricated tungsten. Cracks along the building direction can be clearly observed in Figure 7(a). EBSD results reveal that the microstructure consists of columnar grains (Figure 7(b)), which is slightly different from the strip microstructures in the horizontal cross-sections provided in Figure 6(a) and (b). As has been reported in many articles, columnar grains are formed in SLM due to the direction of heat transfer; they grow toward the molten pool along the *Z*-direction, since the growth velocity is much higher when the crystals growth direction is aligned with the maximum temperature gradient [15,37]. The columnar grain stretching across several layers is favorable for layer bonding. Because, the fracture and defects normally present among layers due to the layerwise building method, the stretched columnar grains could effectively reduce this fabrication orientation-based weakness. Additionally, microcracks are prone to form in the SLM process due to the intrinsic high brittleness of tungsten and the thermal gradients. When high power-density laser irradiates on relative thin powder layer, the high thermal conductivity leads to strong heat flux, which is parallel and negative to the building direction. This heat flux can cause re-melting of underlying layer (as designed in Section 3.1.2), which reduces temperature gradient at the layer interface. Therefore, the thermal stress and microcracks among layers decreased to a great extent. However, it was reported that columnar lamellar microstructures along *Z* direction can provide long unrestricted slip paths for crack growth [38]. In this case, crack can easily initiate and propagate in Z direction, which was verified by the presence of cracks along the building direction in Figure 7(a).

### 3.6. Mechanical performance

**Figure 8.**
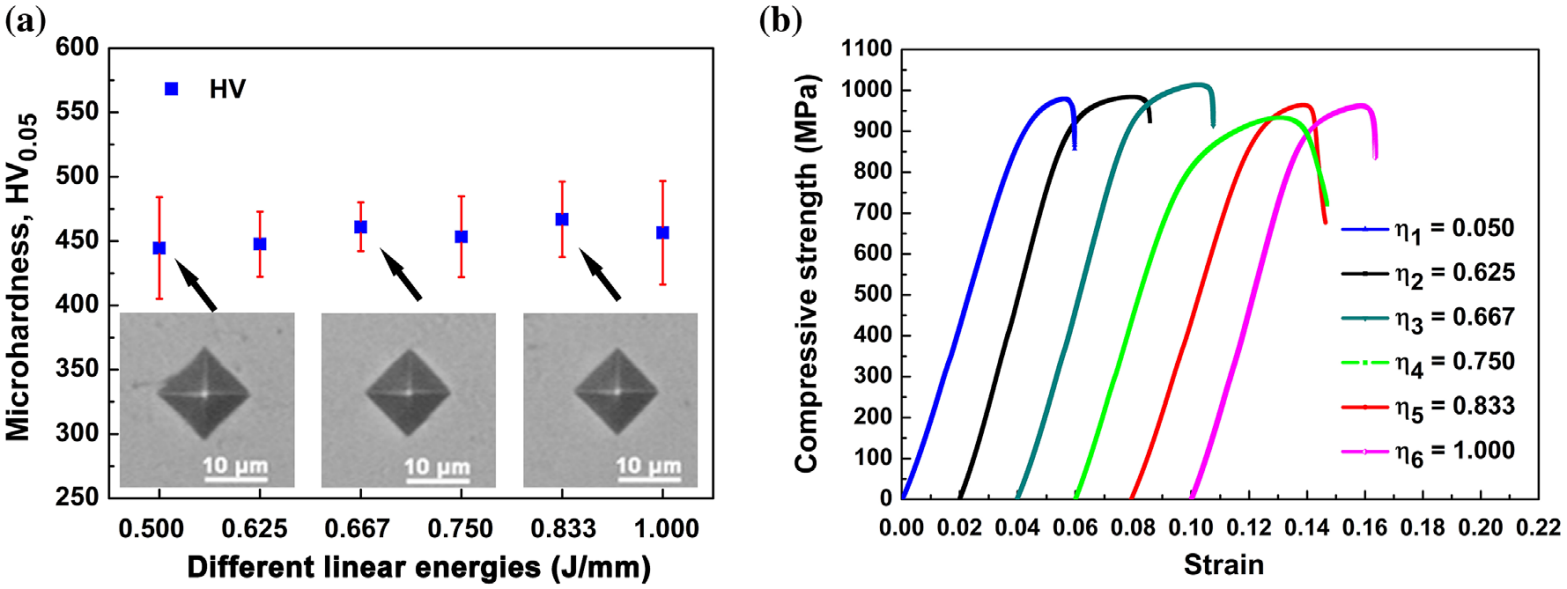
(a) The effect of laser linear energy on microhardness of SLM-processed pure tungsten and (b) compressive stress–strain curves of SLM fabricated pure tungsten with different laser linear energies.

Figure 8(a) illustrates the effect of laser linear energy on microhardness, which was measured from the cross-sections of SLM-processed pure tungsten specimens. The microhardness of the specimens is about 445–467 HV_0.05_, and no statistically signiﬁcant variation of hardness among the different linear energies processed specimens was observed. The hardness is maximal for the *η*_5_ specimen, and exceeds 460 HV_0.05_ for both *η*_3_ and *η*_5_ specimens. The SLM-processed tungsten specimens show a superior hardness compared with conventional PM or SPS processed tungsten (typically 320–400 HV) [39]. The reasons are explained below: the residual stresses in SLM layerwise-build parts are quite large, but they are not always disadvantageous. Because, on the premise of a suﬃciently high densiﬁcation without massive cracks or pores, a reasonable level of residual stress in SLM-processed parts may cause dislocation strengthening and hardness enhancement [7,33,40].

Figure 8(b) shows the compressive stress–strain curves of SLM-processed pure tungsten. The measured mechanical properties are summarized in Table 1. The ultimate compressive strength (UCS) and compressive yield stress (CYS) of *η*_3_ specimen are 1015 and 882 MPa, respectively, which reaches the highest value among all specimens. The *η*_4_ specimen exhibits the lowest compressive strength with the corresponding UCS and CYS of 933 and 791 MPa, respectively. The compressive results are highly consistent with the density results (Figure 5(g)). The mechanical properties of tungsten produced by other conventional fabrication techniques are also listed in Table 2 for comparision. All quoted results were obtained in as-fabricated condition without subsequent treatment and under the same quasi-static compression test. As revealed in Table 2, the compressive stength (especially the *η*_3_) is comparable for SLM and conventional fabrication methods, including CVD, HIP, PM, and SPS. Our results demonstrate that SLM is a new feasible methods for fabrication of pure tungsten parts with desirable performance.

**Table 2.** Comparison of the mechanical properties of pure tungsten fabricated by SLM and conventional processing methods.

Specimens	CYS (MPa)	UCS (MPa)	Strain (%)	Density (g/cm^3^)	*S*_*a*_ (μm)	HV
SLM *η*_1_ = 0.500	868	978	5.97	97.82% (18.88 ± 0.02)	7.59	445 ± 39
SLM *η*_2_ = 0.625	864	984	6.58	98.29% (18.97 ± 0.06)	8.10	448 ± 25
SLM *η*_3_ = 0.667	882	1015	6.76	98.50% (19.01 ± 0.02)	6.74	461 ± 18
SLM *η*_4_ = 0.750	791	933	8.65	97.98% (18.91 ± 0.05)	8.97	452 ± 31
SLM *η*_5_ = 0.833	849	964	6.64	97.93% (18.90 ± 0.03)	7.46	467 ± 29
SLM *η*_6_ = 1.000	860	962	6.36	97.72% (18.86 ± 0.04)	7.44	456 ± 41
CVD [41,42]	–	780–1480	–	≤99.79%	–	419 (4.5 GPa)
HIP [2,43]	1010	1180	–	≤98.00%	–	–
PM [44,45]	900	1000–1200	–	≤98.20%	–	344
SPS [7,39,46]	750	980	–	≤96.30%	–	372 (4 GPa)

## 4. Conclusions

In summary, although, SLM manufacturing of pure tungsten encounters many intractable difficulties due to the material intrinsic properties, we produced high-density thin-wall pure tungsten parts by SLM with optimized processing parameters. The main conclusions were summarized as follows:

(1) Powders in highly spherical shape, sufficiently thin deposition layer thickness, and optimized linear energy were vital controlling factors in SLM-produced tungsten. A reasonable match of these factors was in favor of a high-performance tungsten. Parameter calculation and design prior to experiments could give valuable guidance to the optimization of laser parameters.
(2) Different laser linear energy (*η*) had significant influence on sintering formability, density, and mechanical properties of SLM specimens. In the *η* range of 0.5–3.7 J/mm, the *η*_3_ = 0.667 J/mm presented better sintering formability and performance than the others. Specimens produced with *η*_3_ reached the highest density of 19.01 ± 0.02 g/cm^3^ (98.50 ± 0.12% of theoretical density).
(3) Dense microstructures without balling and with few microcracks were formed in SLM processed pure tungsten. The microcrack in SLM processed pure tungsten seemed inevitable, due to the intrinsic properties of material and the specific processing manner, especially at grain boundary along the building direction. But it seemed not seriously affect the performance. The mechanical properties of SLM-produced tungsten were comparable to conventional fabrication methods, in which the highest hardness and the UCS reached more than 460 HV_0.05_ and 1 GPa, respectively.

The research outcomes suggest that careful tailoring of the laser processing parameters can provide a valuable and cost effective way of optimizing the performance and density of the SLM-produced parts. This research gives us new insights into the application of refractory metals in additive manufacturing.

## Funding

This work was financially supported by the Natural Science Foundation of Guangdong Province [grant number 2016A030312015]; Guangdong Science and Technology Program [grant numbers 2016B090916003; 2017B030314122; 2017A070702016]; Guangzhou Science and Technology Program [grant numbers 201604016109; 201704030111]; Guangdong Academy of Sciences Projects [grant numbers 2016GDASPT-0206; 2017GDASCX-0202; 2017GDASCX-0111; 2018GDASCX-0402].

## Disclosure statement

No potential conflict of interest was reported by the authors.
